# Comparison of Focused Upper Airway Ultrasonographic and Anthropometric Measurements for Predicting Difficult Intubation: A Prospective Observational Study

**DOI:** 10.3390/medicina62071408

**Published:** 2026-07-21

**Authors:** Sami Eksert, Ecem Kahraman, Serdar İnan, Fatih Şimşek, Mehmet Emin İnce, Can Çelik, Gökçe Sezen Aydın, Hasan Soylu, Murtaza Kaya, Gökhan Özkan, Mehmet Burak Eşkin

**Affiliations:** 1 Department of Anesthesiology and Reanimation, Gülhane Training and Research Hospital, University of Health Sciences, General Dr. Tevfik Sağlam Cd. No:1, Etlik, 06010 Ankara, Türkiye; ecem.kahramankara@sbu.edu.tr (E.K.); serdar.inan@sbu.edu.tr (S.İ.); fatih.simsek2@sbu.edu.tr (F.Ş.); can.celik@sbu.edu.tr (C.Ç.); gokcesezen.aydin@sbu.edu.tr (G.S.A.); hasan.soylu@sbu.edu.tr (H.S.); gokhan.ozkan@sbu.edu.tr (G.Ö.); mehmetburak.eskin@sbu.edu.tr (M.B.E.); 2 Department of Emergency Medicine, Kutahya Health Science University, 43000 Kutahya, Türkiye; murtaza.kaya@ksbu.edu.tr

**Keywords:** difficult laryngoscopic view, difficult laryngoscopy, airway ultrasonography, hyoid–skin distance, epiglottis–skin distance, mouth opening, upper lip bite test, body mass index

## Abstract

*Background and Objectives*: The prediction of difficult laryngoscopic view remains an important component of preoperative airway assessment. Conventional bedside tests are widely used, but their diagnostic performance may be limited by subjectivity and interobserver variability. Focused upper airway ultrasonography may provide objective anatomical information and complement standard clinical evaluation. This study aimed to evaluate the diagnostic accuracy of focused upper airway ultrasonographic and conventional anthropometric measurements for predicting difficult laryngoscopic view in adult patients scheduled for tracheal intubation. *Materials and Methods*: This single-center, prospective, observational study included 212 adult patients with American Society of Anesthesiologists physical status I–III who were scheduled for elective surgery under general anesthesia requiring tracheal intubation. Preoperative airway assessment included the Modified Mallampati classification, upper lip bite test, mouth opening, thyromental distance, and sternomental distance. Focused ultrasonographic measurements included hyoid–skin distance and epiglottis–skin distance. Difficult laryngoscopic view was defined as a Cormack–Lehane grade III–IV view recorded during the initial direct laryngoscopic assessment. Diagnostic performance was evaluated using receiver operating characteristic curve analysis, and independent predictors were assessed using multivariable logistic regression. *Results*: Difficult laryngoscopic view was identified in 44 of 212 patients (20.8%). Patients with difficult laryngoscopic view had significantly higher body weight and body mass index, smaller mouth opening, and greater hyoid–skin distance. Hyoid–skin distance was significantly greater in patients with difficult laryngoscopic view than in those without difficult laryngoscopic view (12.00 ± 2.79 mm vs. 10.33 ± 3.16 mm; *p* = 0.002). Mouth opening was significantly smaller in patients with difficult laryngoscopic view (4.39 ± 0.72 cm vs. 4.74 ± 0.99 cm; *p* = 0.030). Epiglottis–skin distance did not differ significantly between groups. In receiver operating characteristic analysis, hyoid–skin distance showed an area under the curve of 0.682, with a cut-off value of ≥12.05 mm, sensitivity of 47.73%, and specificity of 83.33%. In multivariable logistic regression analysis, body mass index and hyoid–skin distance were independently associated with difficult laryngoscopic view, whereas greater mouth opening had a protective effect. The combined multivariable model showed an area under the curve of 0.734; however, at a probability cut-off value of 0.5, its sensitivity was low at 15.9%, despite high specificity of 97.0%. *Conclusions*: Focused upper airway ultrasonography may provide complementary objective information when used together with conventional preoperative airway assessment. Hyoid–skin distance was independently associated with difficult laryngoscopic view and demonstrated relatively high specificity; however, its discriminatory performance was modest and its sensitivity was limited. Therefore, hyoid–skin distance and the combined model should not be interpreted as standalone screening tools for excluding difficult laryngoscopic view. Their main clinical value may be as adjunctive parameters that support risk stratification and advance airway management planning in selected patients.

## 1. Introduction

Airway management is one of the most critical components of perioperative patient safety. Failure to identify a potentially difficult airway before tracheal intubation may result in repeated intubation attempts, airway trauma, inadequate oxygenation, aspiration, and serious cardiopulmonary complications [1,2,3]. Although difficult airway events are relatively uncommon in the general surgical population, their potential consequences are clinically significant. Therefore, accurate and practical preoperative airway assessment remains essential for anticipating airway difficulty and planning an appropriate airway management strategy.

Conventional bedside airway assessment tests, including the Modified Mallampati classification, thyromental distance, sternomental distance, mouth opening, and the upper lip bite test, are routinely used in clinical practice. These tests are simple, inexpensive, and rapidly applicable; however, their predictive performance is limited when used individually. Previous studies and systematic reviews have shown that many conventional tests have variable sensitivity and specificity, and their results may be influenced by patient cooperation, examination technique, and interobserver variability [4,5,6]. Accordingly, there is ongoing interest in objective methods that can complement standard clinical airway assessment.

Difficult laryngoscopic view is an important component of airway difficulty because it may impair glottic visualization and complicate tracheal intubation. However, it should be distinguished from difficult intubation as a broader clinical outcome, which may also include the number of attempts, intubation time, need for adjuncts, optimization maneuvers, alternative devices, or failed intubation. The Cormack–Lehane classification primarily reflects laryngoscopic view rather than all dimensions of clinical intubation difficulty. Therefore, studies using Cormack–Lehane grade III–IV as the outcome should be interpreted as evaluating predictors of difficult laryngoscopic view.

Ultrasonography has emerged as a noninvasive, radiation-free, bedside imaging modality for the evaluation of upper airway anatomy [7,8]. In contrast to conventional clinical tests, airway ultrasonography can provide objective information about anterior neck soft tissue thickness and relevant airway landmarks. Several ultrasonographic parameters, including hyoid–skin distance, epiglottis–skin distance, tongue thickness, tongue volume-related measurements, and pre-epiglottic soft tissue measurements, have been investigated for the prediction of difficult laryngoscopy and difficult laryngoscopic view [9,10,11,12]. However, the literature remains heterogeneous with respect to patient populations, ultrasound probes, measurement planes, definitions of airway difficulty, operator experience, and reported cut-off values.

For an ultrasonographic airway assessment to be clinically useful, especially in routine preoperative practice and time-sensitive airway scenarios, it should be rapid, reproducible, and easy to integrate into standard airway evaluation. Therefore, the present study focused on hyoid–skin distance and epiglottis–skin distance, which are anterior neck midline measurements that can be obtained using a single high-frequency linear probe. These parameters were prioritized because they are noninvasive, rapidly obtainable, based on clearly identifiable anatomical landmarks, and potentially feasible for routine clinical use. In contrast, some other sonographic parameters, such as tongue thickness or tongue volume-related measurements, may require additional acquisition planes, different probe types, or longer examination time, which may reduce practicality in a focused bedside protocol.

The aim of this study was to evaluate the diagnostic accuracy of focused upper airway ultrasonographic measurements and conventional anthropometric parameters for predicting difficult laryngoscopic view in adult patients scheduled for tracheal intubation. Specifically, we assessed the predictive value of hyoid–skin distance and epiglottis–skin distance, together with standard clinical airway tests, and sought to identify practical, objective, and clinically applicable parameters that may contribute to preoperative airway risk stratification.

## 2. Materials and Methods

### 2.1. Study Design and Setting

This was a single-center, prospective, observational study conducted in the Department of Anesthesiology and Reanimation at Health Sciences University Gülhane Training and Research Hospital. Ethics approval was obtained from the Ankara Etlik City Hospital Clinical Research Ethics Committee (Date: 15 October 2025; No: AEŞK-EK-2025-276), which served as the responsible institutional ethics committee for the study site. The study was registered at ClinicalTrials.gov (NCT07309978). The research was conducted in accordance with the Declaration of Helsinki, and written informed consent was obtained from all participants [13]. The manuscript was prepared in accordance with the STROBE checklist [14].

### 2.2. Patient Selection

Adult patients aged 18–99 years with American Society of Anesthesiologists (ASA) physical status I–III who were scheduled for elective surgery under general anesthesia requiring tracheal intubation between 1 November 2025 and 1 February 2026 were included in the study. Exclusion criteria were age younger than 18 years, ASA physical status IV–V, emergency surgery, facial or mandibular deformity, history of tracheostomy, pregnancy or breastfeeding, and the presence of neck pathology that could interfere with ultrasonographic assessment.

The study was conducted as a single-group observational study. No randomization was performed, and no interventional procedure was applied as part of the study. All included patients underwent both conventional anthropometric airway assessment and focused upper airway ultrasonographic evaluation before anesthesia induction.

### 2.3. Data Collection and Conventional Airway Assessment

Demographic data, including age, sex, height, weight, body mass index (BMI), and ASA physical status, were recorded preoperatively. All airway assessments were performed before sedation or anesthesia induction, and no premedication was administered before data collection. To ensure methodological consistency, all assessments were performed according to a predefined standardized measurement protocol.

Conventional airway assessment included the Modified Mallampati classification, upper lip bite test (ULBT), mouth opening, thyromental distance (TMD), and sternomental distance (SMD). The Modified Mallampati classification was assessed with the patient in the sitting position, the head in neutral alignment, and the mouth maximally opened without phonation, according to the visibility of the oropharyngeal structures [15]. The ULBT was used to evaluate mandibular protrusion capacity and was classified as class I when the lower incisors could completely bite the upper lip, class II when the upper lip could be bitten partially, and class III when the lower incisors could not reach the upper lip [16]. Mouth opening was measured as the inter-incisor distance during maximal mouth opening and was recorded in centimeters. TMD was measured as the distance between the thyroid cartilage prominence and the mentum with the head fully extended. SMD was measured as the distance between the manubrium sterni and the mentum with the head fully extended [17].

### 2.4. Focused Ultrasonographic Airway Assessment

Focused upper airway ultrasonographic measurements were performed with the patient in the supine position and the head in neutral alignment. A portable ultrasound device, SonoSite Edge™ (FUJIFILM SonoSite Inc., Bothell, WA, USA), equipped with a 6–13 MHz high-frequency linear probe, was used for all ultrasonographic assessments.

All focused upper airway ultrasonographic measurements were performed by the same final-year anesthesiology resident, who was in the fifth year of anesthesiology training and was familiar with perioperative ultrasonography. The measurements were performed according to a predefined standardized protocol, including fixed anatomical landmarks, probe position, and head–neck position.

The focused ultrasound protocol included two anterior neck measurements: hyoid–skin distance and epiglottis–skin distance. Hyoid–skin distance was defined as the shortest distance between the hyperechoic anterior cortex of the hyoid bone and the skin at the anterior neck midline. Epiglottis–skin distance was defined as the shortest distance between the anterior surface of the epiglottis and the skin at the level of the thyrohyoid membrane [18,19,20].

All ultrasonographic measurements were obtained using a midline approach. Each ultrasonographic measurement was obtained twice during the same examination. After the first measurement, the probe was briefly lifted and repositioned, and a second measurement was then obtained from the same anatomical landmark. The mean of the two measurements was used for statistical analysis. This focused protocol was designed to provide a rapid, standardized, and clinically practical upper airway ultrasound assessment using a single high-frequency linear probe.

**Figure 1:**
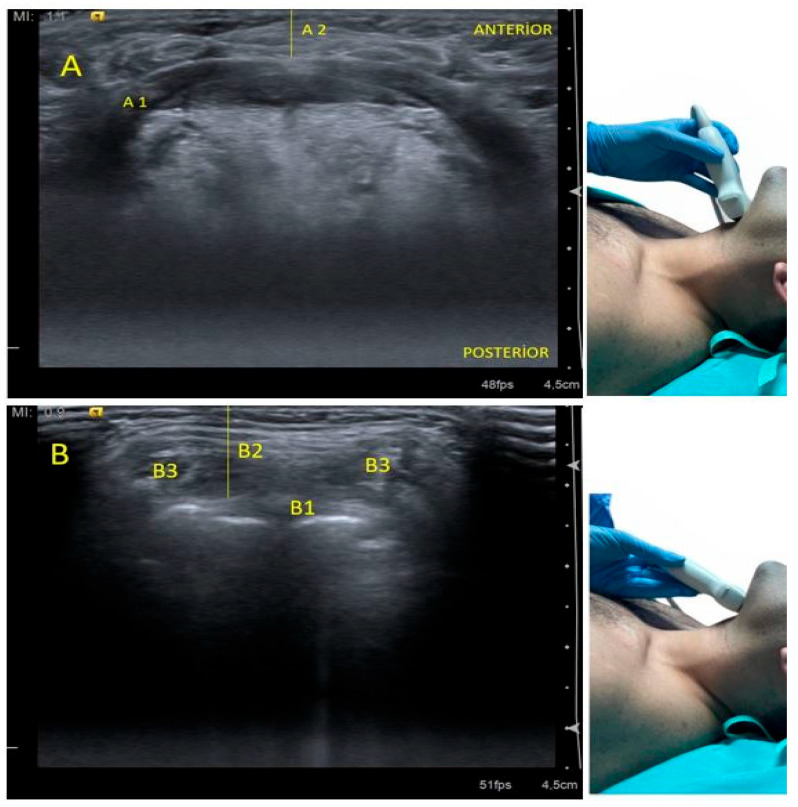
Representative ultrasonographic images demonstrating the measurement technique and anatomical landmarks used for focused upper airway ultrasonography. (**A**) Hyoid–skin distance measured at the anterior neck midline; A1, hyoid bone; A2, skin-to-hyoid distance. (**B**) Epiglottis–skin distance measured at the level of the thyrohyoid membrane; B1, epiglottis; B2, skin-to-epiglottis distance; B3, sternocleidomastoid muscle.

Representative ultrasonographic images demonstrating the standardized acquisition planes and anatomical landmarks for hyoid–skin distance and epiglottis–skin distance measurements are shown in Figure 1.

### 2.5. Study Outcomes

The primary outcome was the diagnostic accuracy of focused ultrasonographic and conventional anthropometric measurements for predicting difficult laryngoscopic view, which was defined as a Cormack–Lehane grade III–IV view recorded during the initial direct laryngoscopic assessment. The secondary outcomes were the identification of independent predictors associated with difficult laryngoscopic view and the evaluation of the predictive performance of a combined clinical and ultrasonographic model.

### 2.6. Anesthesia Induction and Laryngoscopic Assessment Protocol

Standard monitoring was applied to all patients before anesthesia induction. Anesthesia induction was performed with propofol 2 mg/kg, fentanyl 1 µg/kg, and rocuronium 0.6 mg/kg. Intubation was attempted after a 120-s waiting period to allow adequate neuromuscular blockade.

Tracheal intubation was performed by an experienced anesthesiologist using direct laryngoscopy with a Macintosh blade. Laryngoscopic view was evaluated and recorded according to the Cormack–Lehane classification [21]. The anesthesiologist performing direct laryngoscopy and recording the Cormack–Lehane grade was blinded to the preoperative ultrasonographic measurements. The Cormack–Lehane grade was recorded during the initial direct laryngoscopic view, before the use of optimization maneuvers such as external laryngeal manipulation, patient repositioning, or airway adjuncts. In this study, difficult laryngoscopic view was defined as Cormack–Lehane grade III–IV, whereas Cormack–Lehane grade I–II was accepted as non-difficult laryngoscopic view. This definition was used to provide an objective and reproducible endpoint based on laryngoscopic view.

Mask ventilation was assessed according to the Han classification. Grade 1 indicated easy one-person mask ventilation without the need for an adjunct; grade 2 indicated adequate one-person mask ventilation achieved with an oral airway or similar adjunct; grade 3 indicated adequate ventilation achieved only with two-person mask ventilation or marked additional support; and grade 4 indicated failed mask ventilation [22]. The mask ventilation grade was recorded for each patient.

### 2.7. Statistical Analysis

Statistical analyses were performed using IBM SPSS Statistics software version 27.0 (IBM Corp., Armonk, NY, USA). An a priori sample size calculation was performed using G*Power 3.1.9.2. Based on an effect size of 0.99, an alpha error of 0.05, and a statistical power of 0.95, the minimum required sample size was calculated as 58 patients. To improve the precision of estimates and the robustness of multivariable analyses, 212 patients were included in the final analysis.

The distribution of continuous variables was assessed using the Kolmogorov–Smirnov test. Continuous variables were expressed as mean ± standard deviation or median with interquartile range, as appropriate. Categorical variables were expressed as numbers and percentages.

Comparisons between the difficult and non-difficult laryngoscopic view groups were performed using Student’s *t*-test or the Mann–Whitney U test for continuous variables, according to distribution characteristics. Categorical variables were compared using the chi-square test or Fisher’s exact test, as appropriate.

The diagnostic performance of conventional anthropometric and focused ultrasonographic measurements for predicting difficult laryngoscopic view was evaluated using receiver operating characteristic (ROC) curve analysis. The area under the curve (AUC) was calculated, and optimal cut-off values were determined using the Youden index. ROC curves were compared using the DeLong test.

Multivariable logistic regression analysis was performed to identify independent predictors associated with difficult laryngoscopic view. Variables included in the multivariable logistic regression model were selected according to clinical relevance, the previous literature, and the results of between-group comparisons. Because BMI is a composite anthropometric measure derived from body weight and height, BMI was included in the final multivariable model instead of body weight and height to avoid redundancy and preserve model interpretability. The final model included BMI, hyoid–skin distance, mouth opening, and ULBT. Multicollinearity was assessed using variance inflation factor values. Results were reported as odds ratios (ORs) with 95% confidence intervals (CIs). A *p* value < 0.05 was considered statistically significant.

## 3. Results

**Figure 2:**
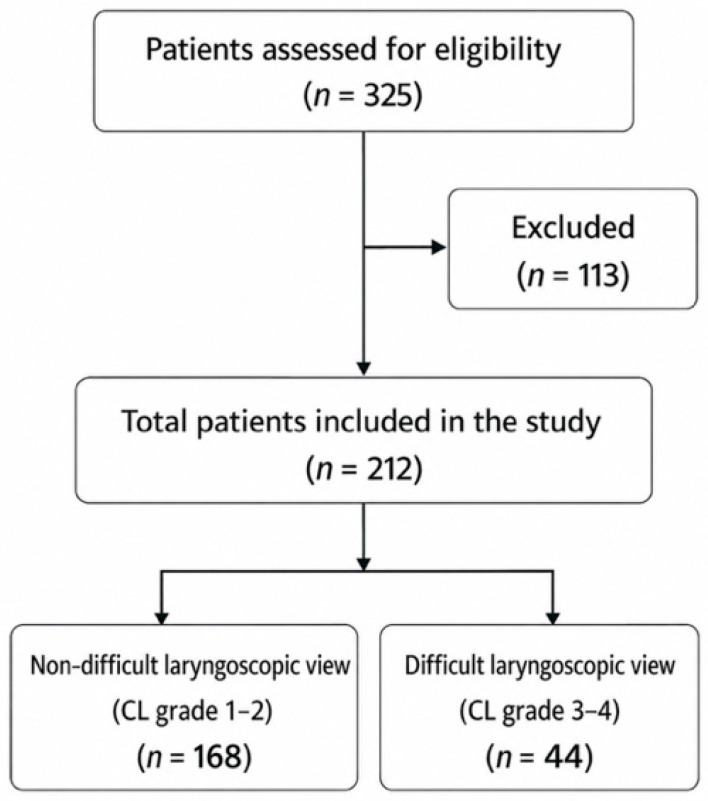
Study flow diagram.

During the study period, 325 patients were assessed for eligibility. A total of 113 patients were excluded according to the predefined exclusion criteria. Of these, 47 underwent regional anesthesia, 33 were managed with a laryngeal mask airway and were therefore not intubated, 15 declined participation, and 18 were excluded because of incomplete data. Consequently, 212 patients were included in the final analysis. Of these, 168 patients had non-difficult laryngoscopic view, defined as Cormack–Lehane grade I–II, and 44 patients had difficult laryngoscopic view, defined as Cormack–Lehane grade III–IV (Figure 2).

There were no statistically significant differences between the non-difficult and difficult laryngoscopic view groups in terms of age, sex, height, or ASA physical status. Mean age was 53.3 ± 15.67 years in the non-difficult laryngoscopic view group and 54.16 ± 9.62 years in the difficult laryngoscopic view group (*p* = 0.739). Sex distribution was also similar between the two groups (*p* = 0.226). In contrast, body weight and BMI were significantly higher in patients with difficult laryngoscopic view. Mean body weight was 83.52 ± 15.87 kg in the difficult laryngoscopic view group and 75.54 ± 13.38 kg in the non-difficult laryngoscopic view group (*p* < 0.001). BMI was also significantly higher in the difficult laryngoscopic view group than in the non-difficult laryngoscopic view group (30.3 ± 4.9 kg/m^2^ vs. 26.5 ± 3.8 kg/m^2^; *p* = 0.002) (Table 1).

When conventional airway assessment tests were evaluated, no statistically significant difference was found between the groups in the Modified Mallampati classification (*p* = 0.153). However, mask ventilation grades according to the Han classification differed significantly between the groups (*p* < 0.001). Grade 3 mask ventilation was more frequent in the difficult laryngoscopic view group than in the non-difficult laryngoscopic view group (40.9% vs. 6.0%). The distribution of the ULBT also differed significantly between the groups (*p* = 0.019). ULBT class III was more common in the difficult laryngoscopic view group than in the non-difficult laryngoscopic view group (9.1% vs. 1.2%), whereas ULBT class I was more frequent in the non-difficult laryngoscopic view group.

Among continuous conventional airway measurements, mouth opening was significantly smaller in the difficult laryngoscopic view group than in the non-difficult laryngoscopic view group (4.39 ± 0.72 cm vs. 4.74 ± 0.99 cm; *p* = 0.030). No statistically significant differences were observed between the groups for sternomental distance or thyromental distance. Mean sternomental distance was 12.29 ± 2.85 cm in the difficult laryngoscopic view group and 12.32 ± 2.83 cm in the non-difficult laryngoscopic view group (*p* = 0.951). Mean thyromental distance was 6.15 ± 1.71 cm in the difficult laryngoscopic view group and 6.49 ± 1.78 cm in the non-difficult laryngoscopic view group (*p* = 0.251) (Table 2).

Among focused ultrasonographic measurements, hyoid–skin distance was significantly greater in the difficult laryngoscopic view group than in the non-difficult laryngoscopic view group (12.00 ± 2.79 mm vs. 10.33 ± 3.16 mm; *p* = 0.002). By contrast, epiglottis–skin distance did not differ significantly between the difficult and non-difficult laryngoscopic view groups (22.23 ± 5.95 mm vs. 21.07 ± 6.40 mm; *p* = 0.283) (Table 2).

**Figure 3:**
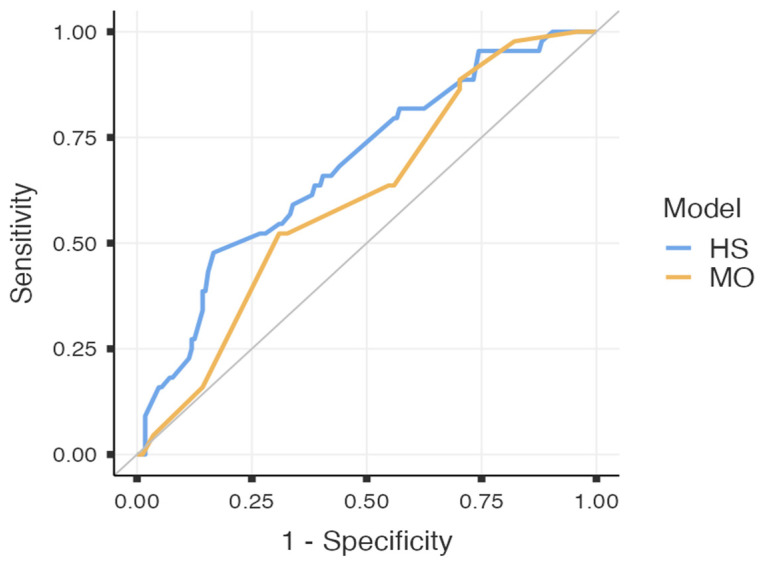
ROC curves of mouth opening and hyoid–skin distance for predicting difficult laryngoscopic view. Abbreviations: HS, hyoid–skin distance; MO, mouth opening. The difference between the AUCs was not statistically significant (DeLong test, *p* = 0.265).

Receiver operating characteristic curve analysis was performed to evaluate the diagnostic performance of mouth opening and hyoid–skin distance for predicting difficult laryngoscopic view. For mouth opening, the optimal cut-off value was ≤4.10 cm. At this threshold, sensitivity was 52.27%, specificity was 69.05%, positive predictive value was 30.67%, negative predictive value was 84.67%, and the AUC was 0.608 (95% CI, 0.521–0.695; *p* = 0.015). For hyoid–skin distance, the optimal cut-off value was ≥12.05 mm. At this threshold, sensitivity was 47.73%, specificity was 83.33%, positive predictive value was 42.86%, negative predictive value was 85.89%, and the AUC was 0.682 (95% CI, 0.595–0.769; *p* < 0.001). Comparison of the ROC curves using the DeLong test showed no statistically significant difference between the AUC values of mouth opening and hyoid–skin distance (*p* = 0.265). However, hyoid–skin distance showed higher specificity than mouth opening (83.33% vs. 69.05%). Nevertheless, its sensitivity was below 50%, indicating that hyoid–skin distance alone would not identify a substantial proportion of patients with difficult laryngoscopic view if used as a standalone screening parameter (Figure 3, Table 3).

A multivariable logistic regression analysis was performed to identify independent predictors associated with difficult laryngoscopic view. The overall model was statistically significant (χ^2^ = 29.0; degrees of freedom = 5; *p* < 0.001), with a Nagelkerke R^2^ of 0.200. In the model, BMI, hyoid–skin distance, mouth opening, and ULBT were evaluated as candidate predictors. BMI was independently associated with difficult laryngoscopic view (OR = 1.1035; 95% CI, 1.02894–1.184; *p* = 0.006). Hyoid–skin distance was also independently associated with difficult laryngoscopic view (OR = 1.1492; 95% CI, 1.02682–1.286; *p* = 0.015). In contrast, greater mouth opening was associated with a lower likelihood of difficult laryngoscopic view (OR = 0.5823; 95% CI, 0.38445–0.882; *p* = 0.011).

For ULBT, no significant difference was observed between class II and class I (OR = 1.2205; 95% CI, 0.58215–2.559; *p* = 0.598). ULBT class III was associated with a higher likelihood of difficult laryngoscopic view compared with class I (OR = 12.0718; 95% CI, 1.52038–95.849; *p* = 0.018). However, this estimate had a wide confidence interval and was based on a small number of patients in ULBT class III; therefore, it should be interpreted with caution (Table 4).

**Figure 4:**
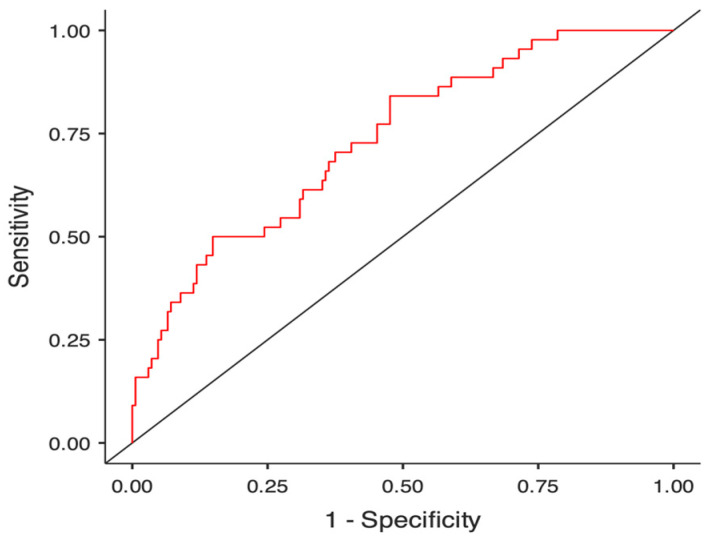
ROC curve of the multivariable logistic regression model for predicting difficult laryngoscopic view. AUC = 0.734. At a probability cut-off value of 0.5, sensitivity was 15.9%, specificity was 97.0%, and accuracy was 80.2%.

The ROC analysis of the prediction curve generated from the multivariable logistic regression model showed an AUC of 0.734. When a probability cut-off value of 0.5 was applied, the model demonstrated an overall accuracy of 80.2%, specificity of 97.0%, and sensitivity of 15.9% for predicting difficult laryngoscopic view. Thus, although the model showed high specificity, its very low sensitivity indicates that it should not be used as a standalone screening tool to rule out difficult laryngoscopic view (Figure 4).

## 4. Discussion

The principal finding of this prospective observational study was that hyoid–skin distance, BMI, and mouth opening were independently associated with difficult laryngoscopic view, defined as a Cormack–Lehane grade III–IV view during the initial direct laryngoscopic assessment. Hyoid–skin distance and BMI were greater in patients with difficult laryngoscopic view, whereas greater mouth opening was associated with a lower likelihood of this outcome. These findings suggest that impaired laryngoscopic visualization may be influenced by both anterior neck soft tissue characteristics and mechanical limitations affecting oral access. However, the outcome evaluated in this study should be distinguished from difficult tracheal intubation as a broader clinical endpoint, which may also include the number of attempts, intubation time, optimization maneuvers, use of adjuncts or alternative devices, and failed intubation.

Among the conventional bedside parameters, mouth opening was significantly smaller in patients with difficult laryngoscopic view than in those without difficult laryngoscopic view (4.39 ± 0.72 cm vs. 4.74 ± 0.99 cm; *p* = 0.030). This finding is clinically plausible because reduced inter-incisor distance directly limits laryngoscope insertion, blade manipulation, and the available working space for tracheal tube placement. In contrast, the Modified Mallampati classification, thyromental distance, and sternomental distance did not differ significantly between the groups. Roth et al. reported that individual bedside airway tests generally have limited diagnostic accuracy when used alone [4]. Similarly, Shiga et al. demonstrated substantial variability in the sensitivity and specificity of conventional airway screening tests [5]. More recently, Wang et al. confirmed that no single bedside examination provides consistently adequate discriminatory performance across adult patient populations [6]. These findings are consistent with the present results and support a multimodal rather than single-test approach to airway assessment.

The distribution of the upper lip bite test also differed between the groups, and ULBT class III was associated with difficult laryngoscopic view in the multivariable model. However, this estimate was based on only six patients and had a wide confidence interval (OR = 12.0718; 95% CI, 1.52038–95.849). Therefore, ULBT class III should not be regarded as a stable independent predictor in isolation. Khan et al. originally described the ULBT as a practical bedside test for assessing mandibular protrusion [16], whereas the systematic review by Faramarzi et al. showed that its diagnostic performance varies across studies and clinical populations [23]. Accordingly, our ULBT finding should be interpreted as supportive and hypothesis-generating and should be considered together with other clinical and ultrasonographic parameters.

Hyoid–skin distance was the most informative focused ultrasonographic measurement in the present study. The mean distance was 12.00 ± 2.79 mm in patients with difficult laryngoscopic view and 10.33 ± 3.16 mm in those without difficult laryngoscopic view (*p* = 0.002). At a cut-off value of ≥12.05 mm, hyoid–skin distance yielded an AUC of 0.682, a sensitivity of 47.73%, and a specificity of 83.33%.

Fernández-Vaquero et al. reported a hyoid–skin distance cut-off of approximately 14.1 mm, with a sensitivity of 80.4% and a specificity of 60.1% [18]. Compared with their findings, our study identified a lower cut-off value and lower sensitivity but higher specificity. This difference may partly reflect methodological variation. Fernández-Vaquero et al. performed the measurements in the sniffing position and used a broader definition of difficult direct laryngoscopy that included Cormack–Lehane grade 2b, whereas our measurements were performed with the head in neutral alignment and the outcome was restricted to Cormack–Lehane grade III–IV.

Yadav et al. reported a sensitivity of 68% and a specificity of 73% for anterior neck soft tissue thickness measured at the hyoid level [19]. In comparison, the hyoid–skin distance in our study showed lower sensitivity but higher specificity. Differences in patient characteristics, measurement technique, head position, and the definition of difficult laryngoscopy may account for this variation.

Tasdemir et al. evaluated an exclusively obese population and reported a mean hyoid–level skin distance of 18.5 ± 3.5 mm in patients with difficult laryngoscopy, together with an AUC of 0.845 [24]. Both the absolute distance and discriminatory performance were greater than those observed in our cohort. This difference may be related to the inclusion of only patients with obesity in their study, because increased regional adipose tissue may result in larger anterior neck measurements and a stronger distinction between difficult and non-difficult laryngoscopic groups.

Ezri et al. found that morbidly obese patients with difficult laryngoscopy had greater pretracheal soft tissue thickness than those with easy laryngoscopy (28.0 ± 2.7 mm vs. 17.5 ± 1.8 mm) [25]. However, their measurement was obtained at the vocal cord level rather than at the hyoid bone. Therefore, absolute values are not directly comparable with those of the present study. Nevertheless, their results support the broader anatomical concept that increased anterior neck soft tissue burden may contribute to impaired glottic visualization.

Although the association between hyoid–skin distance and difficult laryngoscopic view was statistically significant, its diagnostic performance requires cautious interpretation. A sensitivity of 47.73% indicates that more than half of patients with difficult laryngoscopic view would not be identified if this measurement were used alone. Its relatively high specificity suggests that an increased hyoid–skin distance may strengthen an existing clinical suspicion, but a value below the proposed cut-off cannot reliably exclude difficult laryngoscopic view. Therefore, the present findings support the use of hyoid–skin distance as a complementary anatomical measurement rather than as a standalone screening test.

Carsetti et al. concluded that airway ultrasonography may contribute to the prediction of difficult direct laryngoscopy but also identified considerable heterogeneity among studies [9]. Gomes et al. similarly reported marked variation in measurement techniques, cut-off values, and definitions of airway difficulty [10]. Benavides-Zora et al. emphasized that diagnostic performance differs according to the selected sonographic parameter and clinical setting [11], while Giordano et al. noted that the available evidence remains insufficient to support the routine use of any single ultrasonographic measurement [12]. The more recent review by Soni et al. also supported the use of upper airway ultrasonography as an adjunct rather than as a replacement for conventional airway assessment [26]. These conclusions are consistent with the modest AUC and limited sensitivity observed in our study.

In contrast to hyoid–skin distance, epiglottis–skin distance was not significantly associated with difficult laryngoscopic view. The mean distance was 22.23 ± 5.95 mm in the difficult group and 21.07 ± 6.40 mm in the non-difficult group (*p* = 0.283).

Pinto et al. reported that a skin-to-epiglottis distance cut-off of 27.5 mm predicted difficult laryngoscopy with an accuracy of 74.3%, a sensitivity of 64.7%, and a specificity of 77.1% [20]. Their proposed threshold was higher than the mean epiglottis–skin distances observed in both groups in our study. Differences in measurement technique, probe pressure, head position, and patient characteristics may have contributed to the divergent findings.

Fernández-Vaquero et al. reported an epiglottis–skin distance cut-off of approximately 27.0 mm, with a sensitivity of 91.3% and a specificity of 96.9% [18]. These values were substantially higher than the diagnostic performance observed in the present study. Their use of the sniffing position and a broader difficult-laryngoscopy definition may partly explain the difference.

Altınsoy and Bayhan evaluated patients with obesity undergoing bariatric surgery and found that skin-to-epiglottis distance was greater in patients with difficult intubation-related outcomes than in those without difficulty (24.46 ± 3.78 mm vs. 23.06 ± 2.36 mm) [27]. They reported an AUC of 0.954, a sensitivity of 82.5%, and a specificity of 79.45%. These findings differ markedly from the absence of a significant association in our study. However, their study population was restricted to patients with obesity, and their outcome definition incorporated not only laryngoscopic grade but also repeated attempts and the use of additional airway devices. Differences in obesity status, outcome definition, patient position, probe orientation, applied probe pressure, and the exact anatomical plane used for measurement may therefore explain at least part of the variation between studies.

Accordingly, the lack of significance of epiglottis–skin distance in our cohort should not be interpreted as evidence that this parameter is universally uninformative. Rather, its diagnostic contribution appears to be more dependent on the study population and measurement protocol than on hyoid–skin distance.

BMI was significantly higher in patients with difficult laryngoscopic view than in those without difficult laryngoscopic view (30.3 ± 4.9 kg/m^2^ vs. 26.5 ± 3.8 kg/m^2^; *p* = 0.002) and remained independently associated with difficult laryngoscopic view in the final model (OR = 1.1035; 95% CI, 1.02894–1.184; *p* = 0.006). Yuan et al. similarly reported an AUC of 0.746 for BMI and an AUC of 0.732 for neck circumference in the prediction of difficult laryngoscopy [28]. They also identified limited neck mobility as an independent risk factor. Their findings support the relevance of general anthropometric characteristics but also indicate that BMI alone does not fully characterize airway difficulty. Regional anatomy, including anterior neck soft tissue thickness, and mechanical factors, such as mouth opening and mandibular protrusion, should therefore be interpreted together rather than as isolated predictors.

The focused protocol used in the present study was deliberately limited to hyoid–skin and epiglottis–skin distances, both of which can be measured using a single high-frequency linear probe through an anterior midline approach. This design differs from more comprehensive airway ultrasound protocols that include tongue thickness, tongue volume-related measurements, pre-epiglottic space, hyomental distance, or derived ratios. Yadav et al. evaluated both anterior neck soft tissue and tongue thickness [19], while Yao and Wang specifically investigated tongue thickness as a predictor of difficult tracheal intubation [29]. Yuan et al. also assessed multiple upper airway indicators rather than a limited two-parameter protocol [28].

Tongue-related measurements generally require a separate submental acquisition plane and may require a low-frequency convex probe, thereby increasing examination time and procedural complexity. The focused approach may therefore improve feasibility and standardization in routine preoperative practice. Conversely, limiting the examination to two anterior neck measurements may omit other anatomical contributors to difficult laryngoscopic view and may partly explain the modest diagnostic performance observed.

The multivariable model incorporating BMI, hyoid–skin distance, mouth opening, and ULBT showed an AUC of 0.734. Although this value was numerically higher than the AUC values of mouth opening and hyoid–skin distance considered separately, no formal DeLong comparison was performed between the combined model and the individual predictors. Consequently, superiority of the combined model cannot be concluded.

More importantly, at a probability cut-off value of 0.5, the combined model had a specificity of 97.0% but a sensitivity of only 15.9%. This means that the model may identify a small subgroup of patients with a high predicted probability of difficult laryngoscopic view, but it would miss most difficult cases if used as a screening instrument. Therefore, the model should not be used to rule out difficult laryngoscopic view and should not be regarded as a validated clinical prediction tool.

From a clinical perspective, focused upper airway ultrasonography may be most relevant as an adjunct in selected patients with equivocal or borderline bedside findings, obesity, restricted mouth opening, or suspected increased anterior neck soft tissue thickness. It should not replace conventional airway examination or be recommended as a mandatory routine test for all patients on the basis of the present findings.

A brief and standardized ultrasound examination may also have potential value in emergency departments, intensive care units, and other time-sensitive environments. However, the present findings were obtained in elective adult surgical patients undergoing direct laryngoscopy with a Macintosh blade. Extrapolation to critically ill, emergency, pediatric, neonatal, or video laryngoscopy-based airway management therefore requires dedicated validation studies.

### Limitations

This study has several limitations. First, its single-center design and the inclusion of only adult elective surgical patients may limit the generalizability of the findings to emergency, critically ill, pediatric, neonatal, or non-operating room populations. Second, although all ultrasonographic measurements were performed by the same final-year anesthesiology resident using a predefined standardized protocol, formal intraobserver and interobserver reliability analyses were not conducted. Consequently, reproducibility across repeated examinations and across operators with different levels of ultrasound experience remains uncertain. Future multicenter studies should include formal reliability assessment and evaluation of the learning curve required for focused upper airway ultrasonography.

Third, the primary outcome was difficult laryngoscopic view, defined as Cormack–Lehane grade III–IV during the initial direct laryngoscopic assessment, rather than difficult tracheal intubation as a comprehensive clinical endpoint. The number of attempts, intubation time, optimization maneuvers, use of adjuncts or alternative devices, and failed intubation were not evaluated as primary outcomes. In addition, all assessments were performed using direct laryngoscopy with a Macintosh blade before optimization maneuvers, and the focused ultrasound protocol included only two anterior neck measurements. Finally, although 212 patients were included, only 44 difficult laryngoscopic view events were available for five predictor degrees of freedom, resulting in an events-per-variable ratio of approximately 8.8. Together with the absence of formal calibration and external validation, this may increase the risk of overfitting and indicate that the regression findings should be regarded as exploratory and hypothesis-generating.

## 5. Conclusions

Hyoid–skin distance and BMI were independently associated with difficult laryngoscopic view, whereas greater mouth opening was associated with a lower likelihood of this outcome. Hyoid–skin distance demonstrated relatively high specificity but only modest discriminatory performance and limited sensitivity. The combined model also showed high specificity but very low sensitivity and should not be used to exclude difficult laryngoscopic view. Focused upper airway ultrasonography may provide complementary objective information in selected patients but should not replace conventional airway assessment or be regarded as a standalone screening method. Its main potential role is to strengthen clinical suspicion and support advanced airway planning when bedside findings are equivocal or specific risk characteristics are present. Further multicenter studies incorporating reproducibility analysis, calibration, external validation, formal comparison with individual predictors, and broader intubation-related clinical outcomes are required before routine implementation can be recommended.

## Figures and Tables

**Table 1:** Demographic data.

Demographic Data		Non-Difficult Laryngoscopic View (*n* = 168)	Difficult Laryngoscopic View (*n* = 44)	*p* Value
Age (years)		53.3 ± 15.67	54.16 ± 9.62	0.739
Sex	Male	67 (39.9%)	22 (50.0%)	0.226
	Female	101 (60.1%)	22 (50.0%)	
Height (cm)		168.85 ± 9.42	165.86 ± 8.18	0.991
Weight (kg)		75.54 ± 13.38	83.52 ± 15.87	<0.001
BMI kg/m^2^		26.5 ± 3.8	30.3 ± 4.9	0.002
ASA Class, *n* (%)	1	37 (22.0%)	11 (25.0%)	0.366
	2	70 (41.7%)	22 (50.0%)	
	3	61 (36.3%)	11 (25.0%)	

**Table 2:** Comparison of conventional airway assessment tests and focused ultrasonographic measurements.

		Non-Difficult Laryngoscopic View	Difficult Laryngoscopic View	*p* Value
Mallampati Score	1	47 (28.0%)	6 (13.6%)	0.153
	2	62 (36.9%)	20 (45.5%)	
	3	47 (28.0%)	12 (27.3%)	
	4	12 (7.1%)	6 (13.6%)	
Mask Ventilation (Han Classification)	1	86 (51.2%)	11 (25.0%)	<0.001
	2	71 (42.3%)	15 (34.1%)	
	3	10 (6.0%)	18 (40.9%)	
	4	1 (0.6%)	0 (0.0%)	
Cormack–Lehane Classification	Grade 1	81 (48.2%)	0 (0.0%)	Not applicable
	Grade 2	87 (51.8%)	0 (0.0%)	
	Grade 3	0 (0.0%)	37 (84.1%)	
	Grade 4	0 (0.0%)	7 (15.9%)	
Upper Lip Bite Test	1	93 (55.4%)	22 (50.0%)	0.019
	2	73 (43.5%)	18 (40.9%)	
	3	2 (1.2%)	4 (9.1%)	
Mouth Opening (cm)		4.74 ± 0.99	4.39 ± 0.72	0.030
Hyoid–Skin Distance (mm)		10.33 ± 3.16	12.00 ± 2.79	0.002
Epiglottis–skin distance (mm)		21.07 ± 6.40	22.23 ± 5.95	0.283
Sterno-mental distance (cm)		12.32 ± 2.83	12.29 ± 2.85	0.951
Thyro-mental distance (cm)		6.49 ± 1.78	6.15 ± 1.71	0.251

**Table 3:** Diagnostic performance of mouth opening and hyoid–skin distance.

Variable	Cut-Off Value	AUC	Sensitivity/Specificity	PPV/NPV	+LR/−LR	95% CI (AUC)
Mouth opening (cm)	≤4.10	0.608	52.27%/69.05%	30.67%/84.67%	1.687/0.691	0.521–0.695
Hyoid–skin distance (mm)	≥12.05	0.682	47.73%/83.33%	42.86%/85.89%	2.864/0.627	0.595–0.769

**Table 4:** Logistic regression analysis of parameters predicting difficult laryngoscopic view.

Variable	Coefficient (β)	SE	Z	*p* Value	OR	95% CI
Constant (intercept)	−3.4534	1.4168	−2.438	0.015	0.0316	0.00197–0.508
Hyoid–skin distance (mm)	0.1391	0.0575	2.421	0.015	1.1492	1.02682–1.286
Mouth opening (cm)	−0.5409	0.2118	−2.554	0.011	0.5823	0.38445–0.882
Upper lip bite test class II vs. I	0.1993	0.3777	0.528	0.598	1.2205	0.58215–2.559
Upper lip bite test class III vs. I	2.4909	1.0571	2.356	0.018	12.0718	1.52038–95.849
BMI (kg/m^2^)	0.0985	0.0357	2.759	0.006	1.1035	1.02894–1.184

Abbreviations: BMI, body mass index; CI, confidence interval; OR, odds ratio; SE, standard error.

## Data Availability

The data presented in this study are available on request from the corresponding author due to privacy and ethical restrictions.

## Abbreviations

ASA	American Society of Anesthesiologists
AUC	Area under the curve
BMI	Body mass index
CI	Confidence interval
CL	Cormack–Lehane
ESD	Epiglottis–skin distance
HSD	Hyoid–skin distance
LR+	Positive likelihood ratio
LR−	Negative likelihood ratio
NPV	Negative predictive value
OR	Odds ratio
PPV	Positive predictive value
ROC	Receiver operating characteristic
SMD	Sternomental distance
TMD	Thyromental distance
ULBT	Upper lip bite test
US	Ultrasonography
